# The Possible Impact of Antenatal Exposure to Ubiquitous Phthalates Upon Male Reproductive Function at 20 Years of Age

**DOI:** 10.3389/fendo.2018.00288

**Published:** 2018-06-04

**Authors:** Roger J. Hart, Hanne Frederiksen, Dorota A. Doherty, Jeffrey A. Keelan, Niels E. Skakkebaek, Noviani S. Minaee, Robert McLachlan, John P. Newnham, Jan E. Dickinson, Craig E. Pennell, Robert J. Norman, Katharina M. Main

**Affiliations:** ^1^Division of Obstetrics & Gynaecology, University of Western Australia, Perth, WA, Australia; ^2^Fertility Specialists of Western Australia, Bethesda Hospital, Claremont, WA, Australia; ^3^Department of Growth and Reproduction and EDMaRC, Rigshospitalet, University of Copenhagen, Copenhagen, Denmark; ^4^Women and Infants Research Foundation, King Edward Memorial Hospital, Perth, WA, Australia; ^5^Hudson Institute of Medical Research, Monash Medical Centre, Melbourne, VIC, Australia; ^6^Robinson Research Institute, University of Adelaide, Adelaide, SA, Australia

**Keywords:** Raine, endocrine disrupter, testicular volume, testosterone, sperm count

## Abstract

Phthalates are ubiquitous environmental endocrine-disrupting chemicals suspected to interfere with developmental androgen action leading to adverse effects on male reproductive function. Prenatal exposure studies in rodents show cryptorchidism, hypospadias and reduced testicular volume (TV), testosterone and anogenital distance in males. It is postulated that there is a developmental window *in utero* when phthalate exposure has the most potent adverse effects. Some human studies show associations between prenatal phthalate exposure and reduced calculated “free” serum testosterone in infant boys and shorter anogenital distance. However, there are no data available yet which link antenatal exposure to long-term effects in men. We aimed to correlate antenatal phthalate exposure with adult TV, semen parameters and serum reproductive hormone concentrations. 913 men from the Western Australian (Raine) Pregnancy Cohort were contacted aged 20–22 years. 423 (56%) agreed to participate; 404 underwent testicular ultrasound examination; 365 provided semen samples, and reproductive hormones were measured in 384. Maternal antenatal serum phthalate metabolite measurements were available for 185 and 111 men, who provided serum and semen, respectively. Maternal serum collected at 18 and 34 weeks gestation, stored at −80°C, was pooled and analyzed for 32 phthalate metabolites by liquid chromatography-tandem mass spectrometry. TV was calculated, semen analysis performed by WHO approved methods, and serum concentrations of gonadotrophins, inhibin B, and testosterone measured. Eleven phthalate metabolites were detected. Primary and secondary metabolites of di-(2-ethyl-hexyl) phthalate (DEHP) and di-iso-nonyl phthalate (DiNP) were positively correlated. After correction for adult height, BMI, presence of a varicocele and exposure to maternal smoking mono-iso-nonyl phthalate (MiNP) (*r* = −0.22) and sums of DEHP and DiNP metabolites (*r* = −0.24) and the sum of the metabolites of the high molecular weight phthalates (*r* = −0.21) were negatively correlated with TV (all *p* < 0.05). After adjustment for BMI adult serum total testosterone was positively associated with exposure to the following antenatal serum phthalate metabolites: mono-(2-ethylhexyl) phthalate (*r* = 0.26), MiNP (*r* = 0.18), the sum of metabolites for DEHP (*r* = 0.21) and DiNP (*r* = 0.18), and the sum of high molecular phthalates (*r* = 0.20) (*p* = 0.0005 to *p* = 0.02). Given sample size, storage duration and confounding through postnatal exposures, further studies are required.

## Introduction

There is increasing concern about the potential for the environment to impact on reproductive health. We have previously reported that a high proportion of healthy 18- to 20–year-old European and Australian men had semen quality below the WHO reference ranges (1), Jorgensen et al. (2). Furthermore a previous study (2000–2002) of older fertile Australian men (average 35 years) found significantly higher sperm counts than those in our recent study of young Australian men from a birth cohort (3). This raises the question whether a change in semen quality may have occurred in parallel with the increasing incidence of undescended testis, hypospadias and testicular cancer reported in some countries, including Australia (4–8). As registry data of testicular cancer is generally robust, and as the incidence of the disease has increased twofold to fourfold over several generations, it would appear plausible that some form of environmental exposures, or change in lifestyle factors, may have a role in their pathogenesis. It has been suggested that testicular cancer, cryptorchidism, hypospadias and a decrease in semen quality may be symptoms of a testicular dysgenesis syndrome (TDS) due to fetal endocrine disrupter exposures (6). The TDS hypothesis proposes that as a result of abnormal testicular development, a secondary abnormality in Leydig and/or Sertoli cells results during male sexual differentiation, leading to abnormal reproductive development, reduced sperm counts and testicular cancer in later life (7, 9). However, the existence of TDS as a unitary entity has been doubted (10, 11), and a systematic review concluded that, currently there was insufficient evidence to link prenatal exposures with adult male reproductive disorders (12).

Phthalates are diesters of phthalic acid and are widely used in industry and personal care products with exposure through the skin (13), mouth, or through inhalation.^1^ Phthalates are chemicals with endocrine-disrupting properties (14–16) and can cross the placenta to reach the developing fetus (17). They are detectable in amniotic fluid (18), and may reach the newborn *via* breast milk leading to compensated hypogonadism (19). The use of some phthalates [i.e., DnBP, DiBP, BBzP and di-(2-ethyl-hexyl) phthalate (DEHP)], in cosmetics and toys within the European Union, has since been restricted. The TDS hypothesis has some support from animal studies where fetal exposure to phthalates may induce similar features, including dysgenesis of testicles and compensated hypogonadism (20). Additionally, there are reports of a decrease in the anogenital distance (a marker of androgen action), at 2 years of age, in male infants exposed *in utero* to higher levels of phthalate metabolites, as measured from maternal urine during pregnancy (21), although not confirmed by other studies (22, 23). The influence of the phthalate exposure, if significant, may vary according to the timing of the pregnancy exposure (24), and be dose dependent, as differences are not noted in populations with low phthalate exposure (22). However, little is known about the longer-term effects on reproductive health of men after antenatal phthalate exposures. Our study set out to determine any associations between antenatal serum concentrations of phthalate metabolites and markers of testicular function, *via* a correlation analysis, in a cohort of men who have been recognized as a valid reference population (1, 25).

## Materials and Methods

### The Raine Study

The West Australian Pregnancy Cohort (Raine) Study^2^ was designed to measure the relationships between early life events and subsequent health and behavior. The study recruited nearly 3,000 women around 18 weeks gestation in 1989–1991 (26) over a total of 30 months. The 2,868 children (including 1,455 boys) born to 2,804 mothers were retained to form the Raine Study cohort. The cohort is unique because detailed antenatal, postnatal, and childhood measurements have been made. The women originally recruited in pregnancy were equally randomized to an intensive investigation where maternal blood samples were collected at 18 and 34/36 weeks of gestation (and stored in aliquots without thawing at −80°C), and fetal ultrasound measurements were made. A maternal history of cigarette smoking was recorded prospectively at 18 and 34/36 weeks. A general cohort follow-up was undertaken at ages 1, 2, 3, 5, 8, 10, 14, 17, 20, and 22 years with the latest cohort including 1,433 men still alive, making it one of the largest and most closely followed prospective cohorts of pregnancy, childhood, and adolescence in the world. Even at 20 years of age the cohort remained representative of the general Western Australian population (27). Ethical approval was obtained from the University of Western Australia Human Research Ethics Committee, and all participants provided informed written consent.

### Clinical Assessment at 20 Years of Age

All cohort members were invited to attend follow-up, which involved questionnaires, collection of anthropometric data (28), and collection of serum for analysis for testosterone, estradiol (E_2_) and estrone (E_1_), luteinizing hormone (LH), follicle stimulating hormone (FSH), and inhibin B (inhB) concentrations. A testicular ultrasound examination was performed (at King Edward Memorial Hospital) and a semen sample analyzed at the IVF unit (Fertility Specialists of Western Australia) as previously reported (1).

Semen samples were analyzed as per WHO semen manual guidelines (29) including sperm concentrations (million per milliliter), total sperm output (million per ejaculate), and total progressively motile sperm (TMS) being the seminal volume × concentration × motility (%A grade + %B grade) and sperm morphology according to strict WHO criteria. The sperm chromatin structural assay (SCSA) was performed as described (30) with slight modifications. The DNA fragmentation index represents the percentage of sperm within the sample with fragmented or damaged DNA. Serum inhB concentration was measured by Gen II ELISA (Beckman Coulter, Inc., Brea, CA, USA), which had a limit of detection of 2.6 pg/mL. LH and FSH were determined in duplicate by ELISA (IBL International, Hamburg, Germany). The limit of detection of the LH assay was 1.3 IU/L (calibrated against WHO IRP 80/552), while for FSH assay it was 1 IU/L (calibrated against NIBSC 92/510). The intra-assay precision (CV) of the ELISAs ranged from 8 to 11% based on the mean values for low and high value quality control samples from *n* = 16–17 assays. Testosterone, E_2_ and E_1_ were measured by liquid chromatography-tandem mass spectrometry (LC-MS/MS) as described (31), with limits of quantitation for testosterone (0.025 ng/mL), and estradiol (5 pg/mL), respectively.

Testicular ultrasonography was performed as described by a single experienced operator (1), and the volume of each testis calculated by estimating the maximal dimensions in three planes, excluding the epididymis, using electronic calipers and testis volume was calculated using the formula for a prolate ellipsoid (length × width × height × 0.52) (32). One value for length, width and height was recorded per examination, and testicular volume (TV) was recorded as the mean of the right and left testicle. Testicular echogenicity and structures within the scrotum were also assessed including venous diameter measured in the supine position with Valsalva manoeuvre. Varicocele was defined as present when the maximal venous diameter was over 3 mm, and increased with the Valvalva manoeuvre (33).

### Management of Stored Maternal Blood Samples

For each woman, 200 µL aliquots of the 18 and 34 week samples were pooled, frozen, and couriered to Copenhagen, Denmark. A previous pilot study confirmed stability of samples during prolonged storage at either −80°C or up to 15 weeks at −20°C, and demonstrated unaltered recovery when processed without acid addition before storing or after storage (34).

### Phthalate Measurements

Maternal serum samples (*n* = 982), of which 437 were from women pregnant with male fetuses, were analyzed by isotope diluted LC-MS/MS with preceding enzymatic de-conjugation. The method for preparation of serum samples, standard solutions and quality controls, as well as the instrumental analysis and general method validation was based in our previous method (35), and expanded to include 32 metabolites from 15 different phthalate diesters (Table 1; Table S1 in Supplementary Material). The method was modified by using online-TurboFlow-LC-MS/MS technology equipped with a probe for heated electrospray ionization running in negative mode, thereby reducing sample volume to 100 µL. A detailed method description, validation, limits of detections (LOD), linear range, matrix effects, intra-day, and inter-day accuracy and precision are included in Supplemental Materials and Methods and Tables S2–S8 in Supplementary Material for the entire cohort. Samples were analyzed randomly and blinded for the technician in 14 batches, each including calibration standards, 30–40 unknown samples, three blanks, three serum pool controls, and three serum pool controls spiked with native phthalate metabolite standards at low or high level. The inter-day variation, expressed as the relative standard deviation was <20% for all analytes spiked in serum at low level except for MMP, MCPP, mono-iso-nonyl phthalate (MiNP), and MiDP (all ≤ 28%) and <16% for all analytes spiked at high level except for MiDP (20%).

**Table 1:** Phthalate diesters and their respective metabolites detected in maternal serum during pregnancy and sums of metabolites used for statistical analysis.

Phthalate diester		Human serum metabolite	
Di-ethyl phthalate	DEP	Mono-ethyl phthalate	MEP
Di-iso-butyl phthalate	DiBP	Mono-iso-butyl phthalate	MiBP
Di-n-butyl phthalate	DnBP	Mono-n-butyl phthalate	MnBP
		Mono-(3-hydroxybutyl) phthalate	MHBP^a^
Butylbenzyl phthalate	BBzP	Mono-benzyl phthalate	MBzP
Di-(2-ethyl-hexyl) phthalate	DEHP	Mono-(2-ethyl-hexyl) phthalate	MEHP
		Mono-(2-ethyl-5-carboxypentyl) phthalate	MECPP^a^
		Mono-(2-carboxymethyl-hexyl) phthalate	MCMHP^a^
Di-octyl phthalate	DOP	Mono-3-carboxypropyl phthalate	MCPP^a^
Di-iso-nonyl phthalate	DiNP	Mono-iso-nonyl phthalate	MiNP
		Mono-carboxy-iso-octyl phthalate	MCiOP^a^

**Sums of phthalate metabolites**			

ΣMBP (*i* + *n*)	Sum of MiBP and MnBP in ng/mL		
ΣDEHPm	Molar sum of MEHP, MCMHP, and MECPP expressed as DEHP in ng/mL		
ΣDiNPm	Molar sum of MiNP and MCIOP expressed as DiNP in ng/mL		
ΣDEHPm + DiNPm	Molar sum of MEHP, MCMHP, MECPP, MiNP, MCiOP expressed as MEHP in ng/mL		
ΣLMW phth.m	Molar sum of MEP, MiBP, MnBP, and MHBP expressed as MEP in ng/mL		
ΣHMW phth.m	Molar sum of MBzP, MEHP, MCMHP, MECPP, MCPP, MiNP, MCiOP, and MiDP expressed as MEHP in ng/mL		
Σall phth.m	Molar sum of MEP, MiBP, MnBP, MHBP, MBzP, MEHP, MCMHP, MECPP, MCPP, MiNP, MCiOP, and MiDP expressed as MEHP in ng/mL		

*^a^Secondary metabolite, all other metabolites are primary metabolites*.

Individual metabolites and their constructed sums were analyzed; MiBP and MnBP values were combined as the sum of MBP isomers [∑MBP_(_*_i_*_+_*_n_*_)_]. The metabolites mono-(2-ethylhexyl) phthalate (MEHP), MCMHP, and MECPP were combined as the sum of DEHP metabolites (∑DEHPm), and the sum of MiNP and MCiOP as the sum of DiNP metabolites (∑DiNPm). The low molecular weight phthalates MEP, MiBP, MnBP, and MHBP were combined (∑LMW phth.m), and the high molecular weight phthalates (MBzP, MEHP, MCMHP, MECPP, MCPP, MiNP, MCiOP and MiDP) were combined (∑HMW phth.m). In order to sum up the metabolites in nanograms/milliliter the molar concentrations of specific metabolites were calculated, summed, and multiplied by their respective phthalate diester (Table 1) (36).

### Statistical Analysis

All phthalates metabolites values less than the limit of detection (<LOD) were replaced by LOD/√2 (24).

Continuous data were summarized using medians and inter-quartile ranges (reported as Q1 − Q3). Categorical data were summarized using frequency distributions. Where data had a non-Gaussian distribution, power transformation (Box–Cox analysis using Shapiro–Wilks λ as an omnibus estimate) was performed for TV, semen parameters, and reproductive hormone data. Data ranking (five levels) based on the whole cohort was used for highly skewed data (phthalates and abstinence period). The ratio of concentrations of serum hormones LH/T and FSH/InhB were calculated from LH, FSH, T, and inhB raw measurements.

Associations between phthalate metabolites and their sums were performed using Pearson correlation coefficients. Associations between TV, semen parameters, and phthalates were estimated using partial correlation with adjustment for abstinence period, maternal smoking, varicoceles, and adjusted for height in analysis of associations with TV (37). Associations between hormone concentrations and phthalates were estimated using partial correlation with adjustment for BMI.

When significant correlations were found, supplementary Mann–Whitney tests for two independent groups were used for comparisons of sum of phthalates between TV grouped into normal (≥12.6 mL)/abnormal (<12.6 mml) (above and below the lowest 25th centile) and serum testosterone (above and below the lowest 25th centile) to examine differences in the actual phthalate levels.

The study explored associations between the reproductive outcome data and maternal phthalate metabolites without adjustments for multiple testing as our tests only describe differences in the data, and consequently following the recommendations of the American Statistical Association no adjustment for multiple comparisons are required (38, 39). Hypothesis tests were two-sided with *p*-values of <0.05 considered statistically significant (*p* values of ≤0.10 were reported when associations patterns across metabolites were assessed). SPSS (version 22.0, IBM SPSS) statistical software was used for data analysis.

## Results

The study cohort included 913 men who were contactable, of whom 753 participated in the 20-year follow-up study. Of these, 608 completed medical questionnaires, 705 underwent physical examination (including 10 who only provided serum samples), and 423 (56% of those participating in the 20-year follow-up, 46% of contactable men from the original cohort) participated in the testicular function study (Tables 2 and 3). Maternal serum phthalate metabolite concentrations were available for up to 216 males within this testicular cohort overall, including up to 188 participants with serum hormones measurements, and up to 111 participants who also provided semen samples and/or underwent testicular ultrasound examination. There were no differences between the men with and without available maternal serum samples, other than they were slightly older and (of those that had semen for analysis) had better sperm morphology (Table 2). We have previously reported that the men in the Raine cohort who did not take part in the testicular function assessment were very similar to those that did agree to participate, other than being slightly shorter (178 vs 180 cm, *p* = 0.008) and having a lower alcohol consumption (*p* = 0.019) (1).

**Table 2:** Table of flow of participants through the Raine study with respect to availability of maternal antenatal serum for measurement of phthalates, and their involvement in the male reproductive assessment at 20 years of age.

	All Raine study participants	Antenatal phthalates available	No antenatal phthalates available
**Pregnant women enrolled in the study**	2,900	982	1,918
Live births	2,868	953	1,915
Male infants	1,455	437	1,018
Female infants	1,413	516	897
**Male infants**	1,455	437	1,018
Participated in physical examination at 20 year follow-up	705	216	489
Provided blood samples	620	188	432
Participated in reproductive assessment	423	111	312
Provided testicular ultrasound	404	107	297
Provided semen sample	365	101	264

*Participant numbers are shown for each component of the 20-year follow-up; hence univariable or multivariable analyses may result in marginally reduced number of participants due to incomplete data*.

**Table 3:** Anthropometric variables, lifestyle factors, and medical history of the male participants at 20 years of age—comparison between those with and without phthalates data available from maternal serum.

	Phthalates available *n* = 216		Phthalates unavailable *n* = 489		*p-*Value
	*N*	Median (IQR) or *N*(%)	*N*	Median (IQR) or *N*(%)	
Testicular study participation	216	111 (51.4%)	489	312 (63.8%)	0.002*
Testicular assessment	216	107 (49.5%)	489	297 (60.7%)	0.006*
Semen sample	216	101 (46.8%)	489	264 (54.0%)	0.077

**General**					
Age (years)	216	20 (19.8–20.4)	489	19.9 (19.7–20.3)	0.002^b^*
Height (cm)	210	179 (173–185)	477	179 (175–184)	0.629
Height tertile^a^					
Lowest < 176 cm	210	73 (33.8%)	477	151 (30.9%)	0.025*
Middle		57 (26.4%)		179 (36.6%)	
Highest > 182 cm		80 (37.0%)		147 (30.1%)	
Weight (kg)	210	77.2 (68.1–86.3)	477	75.3 (68.4–86.1)	0.859
BMI (kg/m^2^)	210	23.8 (21.4–26.7)	477	23.6 (21.4–26.0)	0.471

**Serum hormones**					
LH (IU/L)	185	10.6 (8.1–12.8)	423	10.4 (8.3–13.2)	0.709
FSH (IU/L)	185	4.3 (2.6–6.2)	423	4.3 (3.0–6.2)	0.288
Inhibin B (pg/mL)	185	215.4 (175.4–262.4)	423	217.2 (167.9–269.0)	0.892
Testosterone (ng/mL)	185	4.5 (3.7–6.1)	422	4.6 (3.6–5.7)	0.680
Testis volume (mean, mL)	106	15.2 (13.1–17.2)	296	14.8 (12.5–17.4)	0.802

**Semen parameter**					
Abstinence (days)	100	2 (2–3)	261	2 (2–3)	0.711
Volume (mL)	101	3 (1.9–3.8)	264	2.7 (1.9–3.7)	0.565
Sperm output (million)	101	113.4 (53.4–193.0)	264	111.5 (49.3–209.9)	0.975
Concentration (million/mL)	101	43 (20.4–75)	264	45 (22.0–69.8)	0.427
Sperm chromatin structural assay %	99	2.9 (1.8–4.8)	259	3.3 (1.8–5.4)	0.933
Morphology (normal, %)	97	6 (4–8)	257	4.5 (3–7)	0.006*
Motility (a + b, %)	101	58 (44.5–67)	260	57 (41.3–66)	0.551
Cryptorchidism	103	1 (0.5%)	292	8 (1.6%)	0.546
Herniorrhaphy	105	1 (0.5%)	291	5 (1.0%)	0.660
Varicocele/s	216	25 (11.6%)	489	77 (15.7%)	0.220
Lifestyle factors^a^					
Maternal smoking	216	38 (17.6%)	461	92 (18.8%)	0.467
Smoker	165	30 (13.9%)	367	54 (11.0%)	0.310

**Alcohol**					
Nil	163	24 (11.1%)		67 (13.7%)	0.530
Moderate		83 (38.4%)		186 (38.0%)	
Binge		56 (12.0%)		113 (23.1%)	

**Illicit drugs**					
Nil	101	73 (33.8%)	262	199 (40.7%)	0.311
Marijuana only		18 (8.3%)		32 (6.5%)	
Other only		5 (2.3%)		14 (2.9%)	
Marijuana and other		5 (2.3%)		17 (3.5%)	

**Medication**					
Nil	101	71 (32.9%)	262	202 (41.3%)	0.302
Non-prescription only		11 (5.1%)		24 (4.9%)	
Prescription only		16 (7.4%)		32 (6.5%)	
Non-prescription and prescription		3 (1.4%)		4 (0.8%)	

*^a^Percentages calculated using 489 and 216 as denominators and may not add to 100% when data are missing*.

*^b^Male participants with phthalates data available tend to be slightly older, in particular at the 95^th^ percentile*.

**p < 0.05.

*LH, luteinizing hormone; FSH, follicle stimulating hormone*.

### Measurement of Antenatal Serum Concentrations of Phthalate Metabolites

In total 32 phthalate metabolites were determined (Table S1 in Supplementary Material); 11 metabolites of six phthalate diesters were detected above LOD (Table 1). Individual phthalate metabolites within either the low or high molecular weight groups were highly correlated, as were the primary and secondary metabolites of DEHP and DiNP (Tables S9 and S10 in Supplementary Material). Eight phthalate metabolites were detectable from more than five mothers and were included in further analysis (Table 4).

**Table 4:** Phthalate metabolites (ng/mL) detected in maternal serum during pregnancy for male participants who had phthalates available (*N* = 216), provided blood samples (*N* = 188) and were in testicular function study (*N* = 111).

		*N* = 216				*N* = 188				*N* = 111			
	LOD (ng/mL)	% > LOD	Min	Median	Max	% > LOD	Min	Median	Max	% > LOD	Min	Median	Max
MEP	0.65	86.1	<LOD	3.88	128.39	85.1	<LOD	3.67	128.39	84.7	<LOD	3.91	128.39
MiBP	0.75	69.4	<LOD	1.38	42.94	68.6	<LOD	1.38	42.94	73.0	<LOD	1.60	42.94
MnBP	0.61	98.1	<LOD	2.97	210.63	97.9	<LOD	2.87	210.63	97.3	<LOD	3.15	210.63
MHBP^a^	0.22	25.9	<LOD	<LOD	2.03	25.0	<LOD	<LOD	1.54	25.2	<LOD	<LOD	2.03
MBzP^a^	0.26	31.0	<LOD	<LOD	5.13	30.3	<LOD	<LOD	5.13	28.8	<LOD	<LOD	5.13
Mono-(2-ethylhexyl) phthalate	0.74	100	1.31	4.12	19.68	100	1.31	4.09	19.68	100	1.31	4.08	19.68
MECPP	0.25	100	0.27	1.06	10.84	100	0.27	1.05	10.84	100	0.28	1.16	10.84
MCMHP	0.39	99.1	<LOD	1.49	16.53	98.9	<LOD	1.50	16.53	100	0.45	1.55	16.53
MCPP^a^	0.19	43.1	<LOD	<LOD	7.58	45.2	<LOD	<LOD	7.58	45.0	<LOD	<LOD	7.58
Mono-iso-nonyl phthalate	0.53	97.2	<LOD	3.94	14.06	97.3	<LOD	4.14	14.06	97.3	<LOD	4.28	14.06
MCiOP	0.13	68.1	<LOD	0.18	15.28	68.6	<LOD	0.18	15.28	73.9	<LOD	0.20	4.12
ΣMBP(*i* + *n*)			<LOD	4.42	253.57		<LOD	4.33	253.57		<LOD	5.08	253.57
ΣDEHPm			2.89	9.86	51.46		2.89	9.69	51.46		4.14	9.85	51.46
ΣDiNPm			<LOD	5.87	28.07		<LOD	6.43	28.04		<LOD	6.48	20.54
ΣDEHPm + DiNPm			2.67	11.46	38.61		2.67	11.46	38.61		4.25	11.95	38.61
ΣLMW phth.m			<LOD	8.91	241.12		<LOD	8.41	241.12		<LOD	8.93	241.12
ΣHMW phth.m			3.81	12.15	38.61		3.81	12.15	38.61		4.25	12.46	38.61
Σall phth.m			9.46	25.17	354.43		9.46	24.77	354.43		9.46	25.49	354.43

*^a^Due to low numbers of detectable samples these are no longer used in subsequent analysis, except for the relevant sums*.

*LOD, limit of detection*.

**Figure 1:**
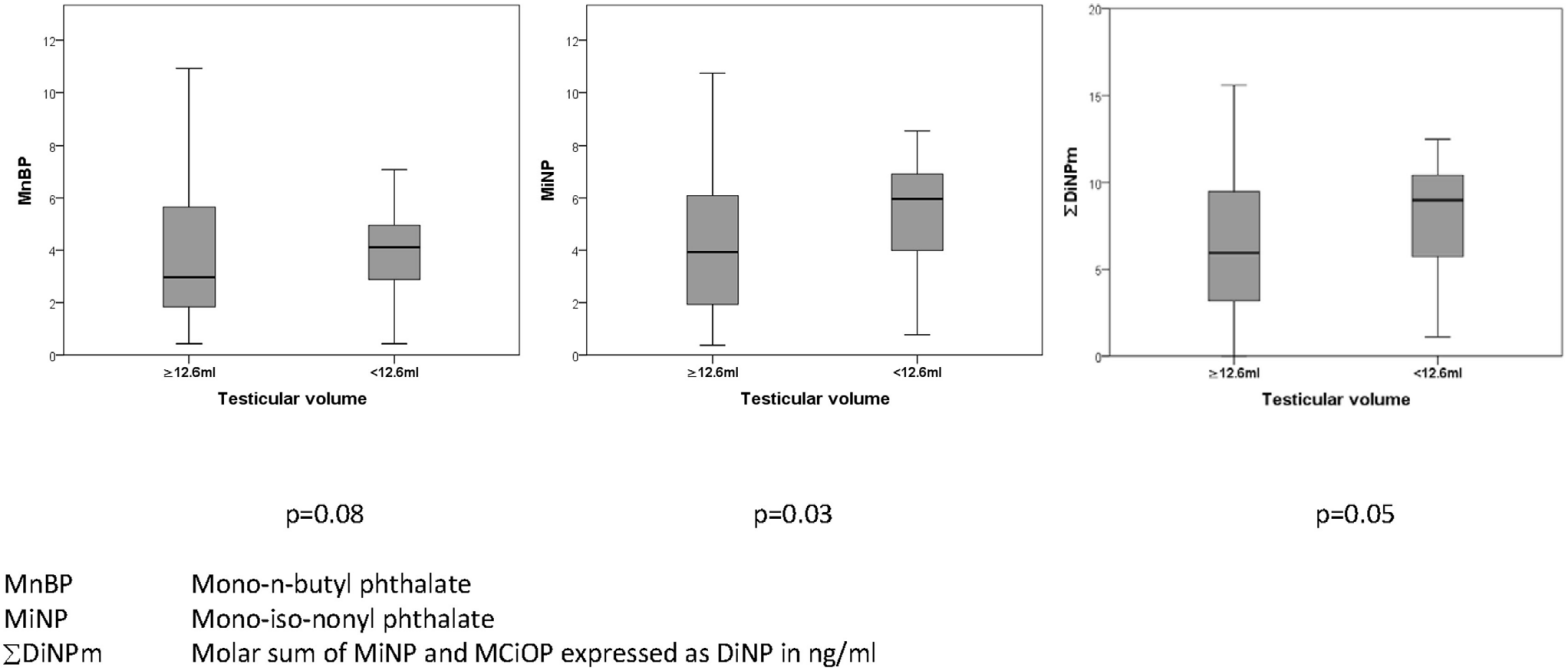
Association of prenatal maternal serum phthalates (nanograms/milliliter) with testicular volume (milliliter) in their adult sons.

Metabolites of the high molecular weight phthalates were inversely associated with TV (−0.21 to −0.24, *p* = 0.016–0.041 with adjustment) (Table 5). The direction of association of all antenatal serum phthalate metabolite concentrations with TV was negative for all other metabolites, apart from MEP, although not significant. In an unadjusted dichotomized analysis of TV below and above the 25th centile (12.6 mL); maternal serum MiNP was associated with a smaller TV: 6.0 (3.7–6.9) vs 3.9 ng/mL (1.9–6.2) (median and inter-quartile range respectively), *p* = 0.03, as was ∑DiNPm: 9.0 (5.4–10.5) vs 5.9 ng/mL (3.2–9.5), *p* = 0.05 (Figure 1). As only two men had cryptorchidism, no association with phthalate exposure was apparent.

**Table 5:** Correlations of adult testicular volume and semen parameters with maternal serum phthalate metabolites.

Ranked phthalates	Testis volume (mL)	Semen sample parameter					
		Volume (mL)	Total sperm output (million)	Concentration (million/mL)	SCSA DFI (%)	Normal morphology %	Motility (*a* + *b* grade)
MEP	0.06	−0.29** (*p* = 0.004)	0.03	0.18*	−0.01	−0.14	0.04
MiBP	−0.11	0.05	0.05	0.00	−0.07	−0.20*	0.14
MnBP	−0.18*	0.04	0.04	0.00	0.05	−0.15	0.11
Mono-(2-ethylhexyl) phthalate	−0.16	−0.02	−0.15	−0.11	0.17*	0.15	−0.12
MECPP	−0.13	−0.06	−0.08	−0.06	0.01	−0.03	−0.10
MCMHP	−0.07	−0.03	−0.07	−0.05	−0.01	−0.02	−0.06
Mono-iso-nonyl phthalate	−0.22** (*p* = 0.033)	0.05	−0.10	−0.14	−0.10	−0.02	0.18*
MCiOP	−0.03	−0.08	−0.09	−0.06	0.03	−0.08	−0.22** (*p* = 0.034)
ΣMBP (*i* + *n*)	−0.19*	0.07	0.01	−0.04	0.01	−0.18*	0.11
ΣDEHPm	−0.16	−0.05	−0.13	−0.10	0.15	0.08	−0.10
ΣDiNPm	−0.20*	0.06	−0.08	−0.12	−0.04	−0.04	0.16
ΣDEHPm + DiNPm	−0.24** (*p* = 0.018)	0.01	−0.14	−0.14	0.05	−0.02	0.05
ΣLMW phth.m	−0.10	−0.10	0.04	0.09	0.02	−0.15	0.12
ΣHMW phth.m	−0.21** (*p* = 0.043)	0.03	−0.12	−0.12	0.06	0.02	0.00
Σall phth.m	−0.15	−0.18*	−0.07	0.02	0.09	−0.15	0.05

*All correlations are adjusted for abstinence period, presence of a varicocele, maternal smoking and BMI at 20 years of age. Testicular volume was also adjusted for adult height *z*-scores (*n* = 91 to 94 for the parameters measured in adulthood)*.

Uncorrected *p*-values are shown as **p < 0.05 and *p < 0.10. No statistically significant correlations were found after adjustment for multiple comparisons (105 = 7 outcomes × 15 phthalates) and no *p*-values < 0.00048 were found.

*SCSA DFI, sperm chromatin structural assay DNA fragmentation index*.

Seminal volume was negatively associated with antenatal serum concentration of MEP (−0.29, *p* = 0.003), and sperm motility with MCiOP concentration (−0.22, *p* = 0.033), after adjustment for abstinence period, presence of a varicocele, maternal smoking and BMI at 20 years of age, in addition TV was also adjusted for adult height (Table 5), but not after adjustment for multiple comparisons. No other systematic or significant associations of antenatal serum concentrations of phthalate metabolites with semen parameters were noted, although the direction of all associations of sperm concentration with antenatal serum phthalate metabolite concentrations were negative, apart from MEP (Table 5).

**Figure 2:**
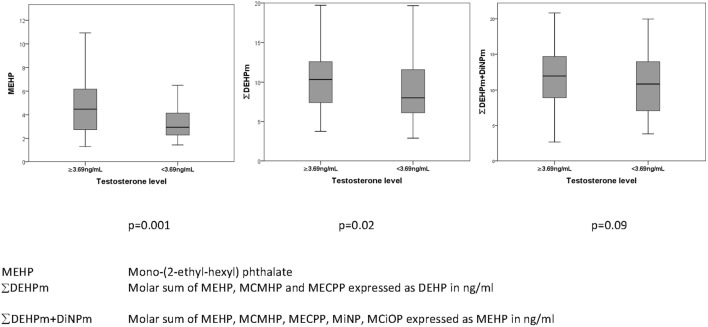
Association of prenatal maternal serum phthalates (nanograms/milliliter) with total serum testosterone (nanograms/milliliter) in their adult sons.

After adjustment for BMI, there were significant positive associations between serum total testosterone concentrations (all *p* < 0.05) and antenatal serum phthalate metabolite concentrations of MEHP, MiNP, ∑DEHPm, ∑DiNPm, ∑HMW phth.m, and ∑DEHPm + DiNPm (Table 6), but not after adjustment for multiple comparisons. In an unadjusted dichotomized analysis of adult serum testosterone above and below the 25th centile (3.69 ng/mL), a higher maternal serum MEHP was associated with a higher adult serum testosterone concentration: 4.5 (2.8–6.3) vs 3.0 ng/mL (2.3–4.4) (median and inter-quartile range), *p* = 0.001, as was ∑DEHPm 10.3 (7.4–12.6) vs 8.0 ng/mL (3.1–11.7), *p* = 0.02 (Figure 2). After adjustment for current BMI, a positive correlation between serum FSH level and maternal serum MiNP concentration (0.14, *p* = 0.049), and serum LH levels and maternal serum ∑DEHPm (0.15, *p* = 0.048) was noted, and a negative association of the serum LH to testosterone ratio with antenatal MEHP concentration (0.19, *p* = 0.0137) (Table 6). No associations between antenatal serum phthalate metabolite concentrations and adult male serum inhB, E_1_, or E_2_ concentrations were found.

**Table 6:** Pearson correlation coefficients between serum hormones and maternal serum phthalate metabolites for 188 male participants, adjusting for BMI at 20 years of age (*n* = 172).

	LH (IU/L)	FSH (IU/L)	InhB (pg/mL)	Testosterone (ng/mL)	LH:T	FSH:InhB	E1 (nmol/L)	E2 (nmol/L)
MEP	0.04	0.06	0.01	0.04	0.00	0.00	−0.11	−0.04
MiBP	0.04	−0.01	−0.07	−0.04	0.06	0.01	−0.04	0.05
MnBP	−0.01	−0.03	−0.01	−0.07	0.05	−0.03	−0.10	−0.02
Mono-(2-ethylhexyl) phthalate	0.06	0.11	0.02	0.26** (*p* = 0.0005)	−0.19** (*p* = 0.01)	0.06	0.05	0.01
MECPP	0.12	0.01	0.03	−0.02	0.10	0.03	0.01	0.08
MCMHP	0.11	0.04	0.05	−0.05	0.13*	0.02	−0.01	0.08
Mono-iso-nonyl phthalate	0.11	0.14** (*p* = 0.0495)	−0.12	0.18** (*p* = 0.01)	−0.08	0.14*	0.02	0.02
MCiOP	−0.04	−0.04	0.02	0.03	−0.06	−0.03	−0.07	0.00
ΣMBP (*i* + *n*)	0.01	−0.02	−0.05	−0.10	0.08	−0.01	−0.08	0.00
ΣDEHPm	0.15** (*p* = 0.048)	0.09	0.08	0.21** (*p* = 0.006)	−0.07	0.04	0.06	0.05
ΣDiNPm	0.09	0.12	−0.13	0.18** (*p* = 0.02)	−0.08	0.12	0.01	0.00
ΣDEHPm + DiNPm	0.13*	0.09	−0.03	0.24** (*p* = 0.001)	−0.11	0.06	0.06	0.03
ΣLMW phth.m	0.06	0.03	0.01	0.06	−0.01	−0.03	−0.07	−0.01
ΣHMW phth.m	0.10	0.10	−0.01	0.20** (*p* = 0.0076)	−0.10	0.07	0.04	0.04
Σall phth.m	0.09	0.06	−0.04	0.11	−0.04	0.05	−0.03	0.05

Uncorrected p-values are shown as **p < 0.05 and *p < 0.10. No statistically significant correlations were found after adjustment for multiple comparisons.

*(120 = 8 outcomes × 15 phthalates) and no *p*-values < 0.00042 were found*.

*LH, luteinizing hormone; FSH, follicle stimulating hormone; T, testosterone; inhB, inhibin B; E1, estradiol; E2, estrone*.

*Ratio of concentrations of serum hormones LH/T and FSH/InhB were calculated from LH, FSH, T, and inhibin raw values*.

*Power transformation was performed for LH, FSH, inhB, Testosterone, E1, and E2*.

*Natural logarithmic transformation was performed for LH:T ratio. Ranking into five groups performed for FSH:InhB ratio*.

## Discussion

This is the first human cohort study to assess whether antenatal serum concentrations of a large range of phthalate metabolites have an association with male reproductive health in early adulthood. We have previously reported within the same cohort that maternal bisphenol A exposure appeared to have no adverse effect of testicular function in adulthood (40). Our data identified a potential association between DEHP and DiNP and TV as well as total serum testosterone. We found a potential positive association between serum FSH concentration and antenatal serum concentration of MiNP, but this was not supported by other DiNP metabolites or any other phthalates, and hence may be a chance finding. Also no effects were evident of serum phthalate metabolites on adult serum inhB or estrogen concentrations. Thus, hormonal associations of antenatal serum phthalate metabolite concentrations were most systematically observed for the LH-testosterone axis. However, given the sample size, potential confounding with additional postnatal exposures and multiplicity of comparisons, further corroboration of these findings is warranted.

Evidence from human and animal studies suggests that our study observations may not be a chance finding. Cross-sectional studies of adult men have demonstrated that current phthalate exposure, as determined by urinary phthalate metabolite excretion, was associated with subtle negative influences in sperm counts and reproductive hormones (41–43). Subtle associations were demonstrated between phthalate monoester levels in breast milk and reproductive hormones in 3-month-old boys, by an increase in the LH: free testosterone ratio and a decrease in free testosterone (19). Phthalates not only affect the hypothalamus-pituitary gonadal axis but also have shown to be associated with serum levels of growth factors and thyroid hormone (44), which in consequence may affect SHBG levels. These complex interactions on several hormone axes in humans may explain the apparent contradiction of results concerning TV and total testosterone in our study. In addition the anogenital distance, which represents an integrated measure of androgen action in humans and rodents, was negatively associated with maternal phthalate exposure during pregnancy (21, 45). First trimester exposures to DBP and high molecular weight phthalates have been correlated with a reduction in maternal free testosterone and an increase in estrogen (46).

Our data are in agreement with some studies in rats which showed that antenatal exposures to phthalates resulted in inhibition of steroidogenesis in Leydig cells leading to development of clinical and histological features similar to TDS in humans. The susceptibility of the rodent testicle to phthalates maybe more pronounced than in the human, and partially reversible (47–49). On the other hand, human exposures are life-long and the patterns of exposures vary with time (50) (see text footnote 1).

Our findings should be viewed cautiously as the study has several important limitations. First, as this is an observational study causality cannot be proven, and we are unable to account for any additional postnatal environmental exposures, which may contribute to the findings (51). Hence, our hypothesis generation lies in the observation that some reproductive parameters showed the same direction of associations with several or all phthalate metabolites. The observed inter-relation of phthalate metabolites is also relevant as phthalates may act additively or synergistically (14, 15). It is proposed that phthalates may exert their endocrine-disrupting effects by inhibition of testosterone and estradiol synthesis (14, 15, 16, 52, 53), aromatase activity inhibition (54), alterations in estrogen metabolism (55–57), and prostaglandin synthesis (58).

A major disadvantage of our study was that only maternal serum samples, and not urine, were available for exposure assessment. This limits the sensitivity of the study (35), as demonstrated by the finding that only 11 of a potential 32 metabolites were detectable in the serum samples, and only eight were present with a frequency sufficient to be used within the analyses. However, given the paucity of the data from human studies, particularly related adult outcomes of antenatal exposures to current environmental chemicals, we believe that our data are relevant and should stimulate further research in this area, and if possible by analyzing maternal urine samples.

It is known that the enzymes involved in hydrolysis of diester phthalates to monoesters are present in blood (59), and may lead to falsely elevated monoester levels after blood sampling if contamination occurs during handling and storage (60). Thus, secondary metabolites are a more reliable measure of true exposure, but they only exist for high molecular weight phthalates. Our laboratory has previously performed a series of pilot studies to ensure that the results of the phthalate analysis of such samples were robust and likely to reflect true phthalate exposure at time of collection (34). In addition, diester contamination is likely to have been relatively minor, since we observed a large inter-individual variation for the first step metabolites, rather than generally high concentrations within the samples. Additionally, it is important to recognize the strong positive correlation between the serum concentrations of the first and second step metabolites of DEHP and DiNP which corroborates our interpretation that the reported MEHP and MiNP concentrations reflect true exposure.

Few previous studies have reported concentrations of phthalate metabolites in serum. The levels of some phthalate metabolites in the present study are in accordance with serum levels observed in others. Median concentrations of MECPP (1.6 ng/mL) and MCiOP (0.2 ng/mL) in men from Greenland, Poland and Ukraine 2002–2004 were comparable to our observations (61). Median levels of MEP (4.1 ng/mL) and MEHP (5.4 ng/mL) from American adults (NHANES 1999–2000) and MEHP (4.3–4.7 ng/mL) in a Swedish population were also similar to our data (62, 63). In contrast, another Danish study of young men found lower levels of MECPP (0.52 ng/mL) and higher levels of MEHP (7.9 ng/mL) (35). The median serum level of all other common phthalate metabolites in the Danish study was below the LOD, while the Swedish study reported higher MEP (11.6 ng/mL) and MiBP (13.4 ng/mL) and the American study higher MnBP (14.4 ng/mL) compared to our data. Despite the potential collection and storage limitations, we thus believe that similar phthalate levels measured in this and other studies are likely to accurately reflect concentrations at the time of collection and true exposure.

In summary, this longitudinal study is the first to explore potential associations between antenatal serum phthalate metabolite concentrations and the reproductive function of young men, in particular with respect to TV and Leydig cell function. Although our findings are of borderline significance, and our study is small, they are plausible and consistent with numerous experimental data and require corroboration. However, due to the study’s limitations, the findings should be viewed with caution until confirmatory data are available from larger, prospectively designed cohort studies.

## Ethics Statement

This study was carried out in accordance with the recommendations of Australian National Health and Medical Research guidelines, and reviewed by the University of Western Australia Human Research Ethics Committee. The protocol was approved by the University of Western Australia Human Research Ethics Committee. All subjects gave written informed consent in accordance with the Declaration of Helsinki.

## Author Contributions

RH conceived the idea for the study, sought funding, coordinated the study and had primary responsibility for writing the manuscript. HF performed all the phthalate analyses and was primarily responsible for writing the supplementary methods and results, and assisted in writing the manuscript. DD was primarily responsible for the statistical analyses and in writing the manuscript. JK organised and supervised the endocrine assays and assisted with data interpretation and writing of the manuscript. NM assisted with statistical analysis and with writing the manuscript. JED was responsible for the testicular ultrasound measurement and assisted with writing the manuscript. NS assisted with data interpretation and with writing the manuscript. RM assisted in data interpretation and with writing the manuscript. RN assisted in data interpretation and with writing the manuscript. CP assisted in writing the manuscript. JN assisted in data interpretation and with writing the manuscript. KM was primarily responsible for the interpretation of the data and assisted with writing the manuscript.

## Conflict of Interest Statement

HF, JK, NM, JED, NS, CP, JN, and KM have nothing to declare. RH is Medical Director of Fertility Specialists of Western Australia, has equity interests in Western IVF, and in the last two years has received grant support from MSD, Merck-Serono, and Ferring Pharmaceuticals. RM has equity interests in the Monash IVF Group. RN has equity interests in FertilitySA, and in the last two years has received grant support from Merck Serono and Ferring Pharmaceuticals.

## Footnotes

^1^www.atsdr.cdc.go. (Accessed: August 1, 2017).

^2^www.rainestudy.org.au. (Accessed: August 1, 2017).
