# Supplementary material for: The Possible Impact of Antenatal Exposure to Ubiquitous Phthalates Upon Male Reproductive Function at 20 Years of Age
Source: Front Endocrinol (Lausanne). 2018 Jun 4;9:288. doi: 10.3389/fendo.2018.00288 (PMC5996240; doi:10.3389/fendo.2018.00288)
Supplement: Supplementary file 1 [file Table_1.PDF]

Supplementary Material

A Possible Impact of Antenatal Exposure to Environmentally Ubiquitous Phthalates Upon Male Reproductive Function at 20 Years of Age.

Roger J. Hart [1,2,8], Hanne Frederiksen [3, 7], Dorota A. Doherty [1,4], Jeffrey A. Keelan [1,4], Niels E. Skakkebaek [3], Novia S. Minaee [4], Robert McLachlan [5], John P. Newnham [1,4], Jan Dickinson [1], Craig E. Pennell [1,4], Robert J. Norman [6], Katharina M. Main [3].

[1] Division of Obstetrics & Gynaecology, University of Western Australia, Perth, WA 6008, Australia.
[2] Fertility Specialists of Western Australia, Bethesda Hospital, 25 Queenslea Drive, Claremont, WA 6010, Australia.
[3] Department of Growth and Reproduction and EDMaRC, Rigshospitalet, University of Copenhagen, Denmark.
[4] Women and Infants Research Foundation, King Edward Memorial Hospital, Perth, Western Australia, Australia.
[5] Hudson Institute of Medical Research, Monash Medical Centre, Melbourne, Australia.
[6] Robinson Institute, University of Adelaide, Adelaide, Australia.
[7] Joint 1st author.

Content:

Supplemental Materials and Methods

Reagents and standards

Preparation of calibration, quality control and validation materials

Analytical method

Operation procedure and method validation

Supplemental Table 1. Phthalate diesters and their respective metabolites analyzed by LC-MS/MS.

Supplemental Table 2. Native and internal (isotope labeled) phthalate metabolite standards, CAS-numbers and suppliers.

Supplemental Table 3. TurboFlow-LC system parameters: mobile phases, solvent gradients and flow-rate.

Supplemental Table 4. Optimized MS/MS interphase settings.

Supplemental Table 5. Phthalate metabolites: Native and internal (labeled) precursor/product ions in negative mode $[M-H]^-$ and retention time (RT), collision energy and tube lens settings for native and internal ions, respectively.

Supplemental Table 6. Matrix effects evaluated by slope and intercept with corresponding 95% confidence intervals (CI) when the responses at 10 concentration levels (range <LOD-100 ng/mL) for phthalate metabolite standards spiked in serum pool were plotted as a function of the responses at same concentration levels for standards prepared in Milli-Q water.

Supplemental Table 7. Method validation: Intra-day· accuracy and precision of quality control materials in Q low and Q high concentrations, limit of detection (LOD) and linear range.

Supplemental Table 8. Method validation: Intra-day precision of quality control materials in Q low and Q high concentrations.

Supplemental Table 9. Correlations between the concentrations of maternal serum phthalate metabolites and their sums.

Supplemental Materials and Methods

Reagents and standards

All native and isotope labeled standards were synthesized by Institut Für Dünnschichttechnologie und Microsensorik (Teltow, Germany) or were purchased from Toronto Research Chemicals (Ontario, Canada) and Cambridge Isotope Laboratory (Andover, MA; distributed by VWR international), **Supplemental Table 2**. Acetone, isopropanol and ammonium acetate were obtained from Merck (distributed by VWR international). Acetonitrile and methanol were obtained from Fisher Scientific (Slangerup, Danmark). 4-methylumbelliferone, 4-methylumbelliferyl-β-D-glucuronide and formic acid were obtained from Sigma-Aldrich (Brøndby, Denmark). β-glucuronidase (Escherichia coli K12) was obtained from Roche Diagnostics (Mannheim, Germany). Milli-Q water was cleaned in a Millipore system (Synthesis A10). All chemicals were of analytical, HPLC or MS grade and all chemicals, solutions and lab wares were controlled for contamination with the actual phthalate metabolites before use. For method validation a serum pool collected randomly among colleagues was used.

Preparation of calibration, quality control and validation materials

A native stock solution containing 100 μg/mL of each native standard in 50% methanol was prepared. Eleven solutions (0.1-500 ng/mL) of the native stock solution solved in 50% methanol were prepared for calibration curves. Furthermore, for determination of limits of detections (LOD) and intra-day variation eight solutions (0.01-50 ng/mL) of the native stock solution were spiked in a randomly collected serum pool (beforehand tested for low or no content of the phthalate metabolites). The serum pool was also used unspiked and spiked at two different levels (Q low and Q high) with the native stock solution for determination of inter-day variation. All calibration and quality control materials were stored at -20°C until use. As solvent blank samples, Milli-Q water was used and treated as all other samples.

Analytical method

The total content of phthalate metabolites (sum of free and conjugated) in serum samples was measured by a newly developed method for simultaneous quantitative determination of 32 different phthalate metabolites (**Supplemental Table 1**) in human serum by isotope dilution TurboFlow-LC-MS/MS with preceding enzymatic deconjugation. After thawing, all serum samples, calibration and validation samples were mixed and aliquots of 100 µL were added 115 µL 1 M ammonium acetate buffer, pH 5.5. Twenty-five µL of internal standard solution (containing 5-40 ng/mL of the different labelled standards dissolved in 50% methanol) and for control of enzyme reaction 50 µL deconjugation mixture (100 ng/mL of 4-methylumbelliferyl β-D-glucuronide and $^{13}C_4$-methylumbelliferone dissolved in 1 M ammonium acetate buffer, pH 5.5) were added to all samples as previously described (Frederiksen et al. 2010). Immediately prior to the incubation for deconjugation all samples were added 10 µL of a freshly prepared enzyme solution (20 v/v % β-glucuronidase dissolved in 1 M ammonium acetate buffer, pH 5.5). The samples were mixed and incubated for 1.5 h at 37°C in a shaking water bath. The enzyme reaction was stopped by adding 50 µL 40% formic acid. To ensure same methanol concentrations in all preparations, the calibration samples (prepared in Milli-Q water added native standards dissolved in methanol) were added 100 µL Milli-Q water, while all control materials (spiked serum pool) and ordinary serum samples were added 50% methanol followed by mixing. Finally, samples were kept in the refrigerator at 4°C followed by 10 min centrifugation at 25,200 g and 4°C. Supernatants were transferred to HPLC vials.

For all extract clean-up, detection and quantification of phthalate metabolites in serum samples, an on-line TurboFlow-LC-MS/MS system (Thermo scientific Aria TLX-1 LC system coupled to TSQ Ultra triple quadrupole mass spectrometer from Thermo Fisher Scientific, San Jose, CA, USA) was used in combination with Aria operating software 1.6.3 and Xcalibur 2.1.0.1139 system software (ThermoFinnigan, Bellefonte, PA, USA). For sample extraction and chromatographic separation of analytes the TurboFlow-LC system was equipped with TurboFlow Cyclone P columns

(0.5 x 50mm) and Hypersil Gold aQ columns (4 x 50 mm, 3 µm particle size) (both from Thermo Scientific, Franklin, MA, USA). The MS/MS-system was equipped with a heated electrospray ionization source (HESI) and all samples were analyzed in negative mode. All sample batches were kept on the auto-sampler at 10°C. The injection volume was 100 µL with flow rate and solvent programming as shown in **Supplemental Table 3**. The optimized MS/MS interphase settings used are shown in **Supplemental Table 4** and the MS transitions, retention times, collision energies and S-lens settings optimized for each single analyte are shown in **Supplemental Table 5**.

Operation procedure and method validation

Matrix effect and ion suppression were investigated in duplicate calibration curves in Milli-Q water and serum pool at 8-10 concentration levels for each compound (0.1-50 ng/mL). The responses from standards prepared in the serum pool were plotted as a function of the responses from samples prepared in Milli-Q water. Subsequently, 95% confidence intervals were calculated for slopes and intercepts of the linear regression using the regression function in Analysis Toolpak for Microsoft Excel 2007. If the 95% confidence intervals included 1 for the slopes and 0 for the intercepts, no matrix effect was present. In cases where e.g. slopes for calibration curves made in serum and water differed (matrix effects) or where e.g. internal standard was different from the specific native analyte and thereby changed the slope of the calibration curves prepared in water from calibration curves prepared in serum, results were corrected for this matrix effect by dividing with the slope coefficient observed in calibration curves made in the serum pool (**Supplemental Table 6**).

For calibration curves, the ratio between the area of native standard and internal standard was plotted as a function of concentration of native standards. By linear regression based on area ratios (sample area/internal standard area) the concentration of unknown samples and the control material were determined.

For method validation and all other analysis two calibration curves in Milli-Q water were included at the beginning and the end of all sample batches. Furthermore, five calibration curves made in serum pool were used for estimation of the intra-day variability: Accuracy (% recovery) and precision (relative standard deviation (RSD)) were calculated for a low and a high concentration level (Q Low and Q High) of the five repeated calibration curves made in the serum pool (**Supplemental Table 7**). The five repeated calibration curves made in serum pool were used for determination of linearity and limit of detection (LOD). For this the approach described by the International Conference on Harmonisation of Technical Requirements for Registration of Pharmaceuticals for Human Use (ICH) guidelines (ICH, 2005) on validation of analytical procedures was used: 3.3 times the standard error of the intercept of the calibration curve with the y-axis divided by the slope of the calibration curve using the five lowest calibration levels for each analyte (Frederiksen et al., 2013; Soeborg et al., 2013). The standard error of the intercept and the slope of the calibration curve were calculated using the regression function in Analysis Toolpak for Microsoft Excel 2007 (Microsoft Corp., Redmond, WA).

The inter-day variation (precision) was estimated from analysis of control material; quality control material was analyzed in triplicates in 14 batches over a period of three month (**Supplemental Table 8**).

Supplemental references

Frederiksen H., Jørgensen N. and Andersson A.-M. (2010) Correlations between phthalate metabolites in urine, serum and seminal plasma from young Danish men determined by isotope dilution liquid chromatography tandem mass spectrometry. *J Anal Toxicol.* 49, 400-410

Frederiksen H., Aksglaede L., Sorensen K., Nielsen O., Main K.M., Skakkebæk N.E., Juul A., Andersson A.-M. (2013) Bisphenol A and other phenols in urine from Danish children and adolescents. *Int J Hygiene and Env Health*. 216, 710-720

Søeborg T., Frederiksen H., Johansen T.H., Fruekilde P., Juul A. and Andersson A.M. (2013) Serum concentrations of DHEA, DHEAS, 17α-hydroxyprogesterone, Δ4-androstenedione and testosterone in children determined by TurboFlow-LC-MS/MS. *Clin Chem Acta*. 419, 95-101

Supplemental Table 1. Phthalate diesters and their respective metabolites analyzed by LC-MS/MS

Phthalate diester	Abbreviation	Human serum metabolite	Abbreviation	LOD* (ng/mL)
Di-methyl phthalate	DMP	Mono-methyl phthalate	MMP	0.44
Di-ethyl phthalate	DEP	Mono-ethyl phthalate	MEP	0.65
Di-iso-propyl phthalate	DiPrP	Mono-(4-oxopentyl) phthalate	MiPrP	0.4
	DPrP	Mono-propyl phthalate	MPrP	0.23
Di-iso-butyl phthalate	DiBP	Mono-iso-butyl phthalate	MiBP	0.75
Di-n-butyl phthalate	DnBP	Mono-n-butyl phthalate	MnBP	0.61
		Mono-(3-hydroxybutyl) phthalate	MHBP	0.22
Butylbenzyl phthalate	BBzP	Mono-benzyl phthalate	MBzP	0.26
Di-n-pentyl phthalate	DPP	Mono-n-pentyl phthalate	MPP	0.27
		Mono-(4-hydroxypentyl) phthalate	MHPP	0.38
Di-(2-ethyl-hexyl) phthalate	DEHP	Mono-(2-ethyl-hexyl) phthalate	MEHP	0.74
		Mono-(2-ethyl-5-hydroxyhexyl) phthalate	MEHHP	0.59
		Mono- (2-ethyl-5-oxohexyl) phthalate	MEOHP	0.45
		Mono-(2-ethyl-5-carboxypentyl) phthalate	MECPP	0.25
		Mono-(2-carboxymethyl-hexyl) phthalate	MCMHP	0.39
Di-n-hexyl phthalate	DHxP	Mono-n-hexyl phthalate	MHxP	0.38
		Mono-(5-hydroxyhexyl) phthalate	MHHxP	0.26
		Mono-(5-carboxypentyl) phthalate	MCPeP	0.2
Di-cyclohexyl phthalate	DCHP	Mono-cyclohexyl phthalate	MCHP	0.27
Di-n-heptyl phthalate	DHpP	Mono-n-heptyl phthalate	MHepP	0.38
		Mono-(6-hydroxyheptyl) phthalate	MHHpP	0.15
		Mono-(6-carboxyhexyl) phthalate	MCHxP	0.23
Di-octyl phthalate	DOP	Mono-octyl phthalate	MOP	0.7
		Mono-3-carboxypropyl phthalate	MCPP	0.19
Di-iso-nonyl phthalate	DiNP	Mono-iso-nonyl phthalate	MiNP	0.53
		Mono-hydroxy-iso-nonyl phthalate	MHiNP	0.4
		Mono-oxo-iso-nonyl phthalate	MOiNP	0.31
		Mono-carboxy-iso-octyl phthalate	MCiOP	0.13
Di-iso-decylphthalate	DiDP	Mono-iso-decyl phthalate	MiDP	0.72
		Mono-(9-hydroxydecyl) phthalate	MHiDP	0.31
		Mono-(9-oxodecyl) phthalate	MOiDP	0.31
		Mono-(9-carboxynonyl) phthalate	MCiDP	0.32

*LOD, Limit of detection (according to ICH algorithm) based on phthalate standards spiked in serum pool

Supplemental Table 2. Native and internal (isotope labeled) phthalate metabolite standards, CAS-numbers and suppliers

Metabolite	Cas-numbers	Supplier	Internal standard	Supplier
MMP	4376-18-5	CIL	$^{13}C_2$-MMP	CIL
MEP	2306-33-4	CIL	$^{13}C_2$-MEP	CIL
MiPrP	35118-50-4	CIL	$^{13}C_2$-MEP	CIL
MPrP	4376-19-6	IDM	$^{13}C_2$-MEP	CIL
MiBP	30833-53-5	CIL	D_4-MiBP	IDM
MnBP	131-70-4	CIL	$^{13}C_2$-MnBP	CIL
MHBP	57074-43-8	TRC	D_4-MHBP	TRC
MBzP	2528-16-7	CIL	$^{13}C_2$-MBzP	CIL
MPP	24539-56-8	CIL	D_4-MPP	IDM
MHPP		IDM	$^{13}C_2$-MCPP	CIL
MEHP	4376-20-9	CIL	$^{13}C_2$-MEHP	CIL
MEHHP	40321-99-1	CIL	$^{13}C_4$-MEHHP	CIL
MEOHP	40321-98-0	CIL	$^{13}C_4$-MEOHP	CIL
MECPP	40809-41-4	CIL	D_4-MECPP	IDM
MCMHP	82975-93-7	CIL	$^{13}C_4$-MCMHP	CIL
MHxP	24539-57-9	IDM	D_4-MHxP	IDM
MHHxP		IDM	D_4-MHHxP	IDM
MCPeP		IDM	D_4-MCPeP	IDM
MCHP	7517-76-4	TRC	$^{13}C_2$-MCHP	CIL
MHepP		IDM	D_4-MHepP	IDM
MHHpP		IDM	D_4-MHHpP	IDM
MCHxP		IDM	D_4-MCHxP	IDM
MOP	5393-19-1	CIL	$^{13}C_2$-MOP	CIL
MCPP	66851-46-5	CIL	$^{13}C_2$-MCPP	CIL
MiNP	519056-28-1	CIL	$^{13}C_2$-MiNP	CIL
MHiNP		IDM	D_4-MHiNP	IDM
MOiNP	936022-00-3	IDM	D_4-MOiNP	IDM
MCiOP	898544-09-07	IDM	D_4-MCiOP	IDM
MiDP	297182-84-4	CIL	D_4-MiDP	IDM
MHiDP		IDM	D_4-MHiDP	IDM
MOiDP		IDM	D_4-MOiDP	IDM
MCiNP		IDM	D_4-MCiNP	IDM
4-MB	90-33-5	Sigma	$^{13}C_4$-4MB	CIL

TRC: Toronto Research Chemicals; CIL: Cambridge Isotope Laboratory; IDM: Institut Für Dünnschichttechnologie und Microsensorik (specific synthesis)

Supplemental Table 3. TurboFlow-LC system parameters: mobile phases, solvent gradients and flow-rate.

	TurboFlow-column/loading pump[a]							Analytic column/eluting pump[b]			
**Step**	**Time (min)**	**Flow (µl/min)**	**% A**	**% B**	**% C**	**Tee**	**Loop**	**Flow (µl/min)**	**Gradient**	**% A**	**% B**
1	0.00	0.3	100			-	out	0.7	step	95	5
2	1.00	0.1	100			T	in	0.6	step	95	5
3	2.00	1.5		100		-	in	0.7	step	95	5
4	2.48	1.5		100		-	in	0.7	ramp	75	25
5	2.50	1.5			100	-	in	0.7	ramp	74	26
6	3.50	0.2	20	80		-	in	0.7	ramp	73	27
7	4.50	0.2	100			-	out	0.7	ramp	62	38
8	15.00	0.2	100			-	out	0.7	ramp	55	45
9	16.25	0.2	100			-	out	0.7	ramp	50	95
10	16.85	0.2	100			-	out	0.7	ramp	20	80
11	17.85	0.2	100			-	out	0.7	ramp		100
12	19.85	0.2	100			-	out	0.7	step		100
13	21.35	1.5	100			-	out	0.7	step	95	5
14	21.37	1.5	100			-	out	0.7	step	95	5
15	23.37	1.5	100			-	out	0.7	step	95	5

[a] Mobile phases for loading pump: A, 10 mM ammonium acetate; B, 0.1% formic acid in methanol; C, acetone/isopropanol/acetonitril (10:45:45)

[b] Mobile phases for eluting pump: A, water; B, methanol

Supplementary table 4. Optimized MS/MS interphase settings

	**Negative mode**
Spray Voltage (V)	3500
Discharge current (V)	4
Vaporizer temperature (°C)	400
Capillary temperature (°C)	220
Sheath gas (N_2) pressure (units)	40
Auxiliary gas (N_2) pressure (units)	15
Ion sweep gas (N_2) pressure (units)	0
Collision gas (Ar) pressure (mTorr)	1.5
Declustering voltage (V)	5
Scan type	SRM
Chrome filter peak width (s)	3
Scan time (s)	0.8
Skimmer Offset (V)	5
Peak with for Q1 (Da)	0.7
Peak with for Q3 (Da)	0.7
MS run time (min)	19.0
Divert valve settings (min)	
1. injection to waste	0-0.15
2. injection to TSQ	0.15-18.7
3. injection to waste	18.7-19.0

SRM, selective reaction monitoring

Supplemental Table 5. Phthalate metabolites: Native and internal (labeled) precursor/product ions in negative mode $[M-H]^-$ and retention time (RT), collision energy and tube lens settings for native and internal ions, respectively.

Native standard	Precursor/product ions m/z	Internal standard	Labeled precursor/product ions m/z	Retention time min	Collision Energy eV	T-Lens V
MMP	179 → 77	$^{13}C_4$-MMP	183 → 79	1.99	24, 23	108, 114
MEP	193 → 77	$^{13}C_4$-MEP	197 → 79	2.73	19, 18	130, 105
MiPrP*	207 → 77			3.81	19	119
MPrP*	207 → 77			4.16	19	119
MiBP	221 → 77	D_4-MiBP	225 → 81	6.29	20, 20	111, 106
	221 → 134			6.29	17	104
MnBP	221 → 77	$^{13}C_4$-MnBP	225 → 79	6.68	20, 20	111, 103
MHBP	237 → 121	D_4-MHBP	241 → 125	2.07	20, 20	109, 124
MBzP	255 → 183	$^{13}C_4$-MBzP	259 → 186	7.87	15, 15	112, 119
MPP	235 → 77	D_4-MPP	239 → 81	10.11	21, 22	129, 129
MHPP**	251 → 121			2.49	23	111
MEHP	277 → 134	$^{13}C_4$-MEHP	281 → 137	16.14	19, 18	134, 120
MEHHP	293 → 121	$^{13}C_4$-MEHHP	297 → 124	6.5	23, 23	118, 146
MEOHP	291 → 121	$^{13}C_4$-MEOHP	295 → 124	6.75	21, 20	141, 133
MECPP	307 → 159	$^{13}C_4$-MECPP	311 → 159	6.36	17, 15	112, 120
	307 → 121			6.36	17	112
	307 → 113			6.32	17	112
MCMHP	307 → 159	$^{13}C_4$-MCMHP	311 → 159	8.05	17, 15	105, 95
MHxP	249 → 77	D_4-MHxP	253 → 81	14.01	20, 28	118, 124
MHHxP	265 → 121	D_4-MHHxP	269 → 125	3.26	19, 20	154, 122
MCPeP	279 → 131	D_4-MCPeP	283 → 131	3.3	17, 15	109, 114
MCHP	247 → 97	$^{13}C_2$-MCHP	251 → 97	9.09	19, 19	126, 126
MHepP	263 → 77	D_4-MHepP	267 → 81	15.87	23, 27	123, 127
MHHpP	279 → 121	D_4-MHHpP	283 → 125	4.48	21, 21	128, 124
MCHxP	293 → 145	D_4-MCHxP	297 → 145	4.59	17, 17	106, 106
MOP	277 → 127	$^{13}C_4$-MOP	281 → 127	16.31	20, 19	120, 127
MCPP***	251 → 103	$^{13}C_4$-MCPP	255 → 103	2.14	58, 58	96, 106
MiNP	291 → 77	$^{13}C_4$-MiNP	299 → 79	16.3	29, 29	140, 154
MHiNP	307 → 121	D_4-MHiNP	311 → 125	8.49	17, 23	112, 139
MOiNP	305 → 121	D_4-MOiNP	309 → 125	8.92	20, 23	128, 128
MCiOP	321 → 173	D_4-MCiOP	325 → 173	8.47	20, 18	122, 117
MiDP	305 → 77	D_4-MiDP	309 → 81	16.79	29, 29	137, 137
MHiDP	321 → 121	D_4-MHiDP	325 → 125	11.72	23, 22	121, 121
MOiDP	319 → 121	D_4-MOiDP	323 → 125	12.56	22, 23	125, 130
MCiNP	335 → 187	D_4-MCiNP	339 → 187	11.71	20, 18	113, 115
4-MB	175 → 133	$^{13}C_4$-4MB	179 → 135	2.47	24, 23	110, 110

*13C4-MEP was used as internal standard

**13C4-MCPP was used as internal standard

***Could also be a metabolite of DnBP and other phthalate diesters

Supplemental Table 6. Matrix effects evaluated by slope and intercept with corresponding 95% confidence intervals (CI) when the responses at 10 concentration levels (range <LOD-100 ng/mL) for phthalate metabolite standards spiked in serum pool were plotted as a function of the responses at same concentration levels for standards prepared in Milli-Q water.

	**Slope**	**95% CI**		**Intercept**	**95% CI**	
**Metabolite**	**mean**	**lower**	**upper**	**mean**	**lower**	**upper**
MMP	0.813	0.804	0.823	**0.005**	**-0.001**	**0.011**
MEP	**1.016**	**0.979**	**1.052**	**0.002**	**-0.005**	**0.009**
MiPrP	0.934	0.895	0.972	**0.004**	**-0.002**	**0.010**
MPrP	0.917	0.872	0.963	**0.003**	**-0.006**	**0.011**
MiBP	**1.010**	**0.982**	**1.038**	**0.000**	**-0.004**	**0.005**
MnBP	**0.977**	**0.945**	**1.010**	**0.006**	**0.000**	**0.013**
MHBP	-0.078	-0.713	0.558	**0.442**	**-0.055**	**0.938**
MBzP	0.194	-0.456	0.843	**0.384**	**-0.123**	**0.891**
MPP	0.966	0.950	0.981	**0.003**	**-0.008**	**0.014**
MHPP	**0.948**	**0.894**	**1.002**	**0.009**	**-0.039**	**0.058**
MEHP	1.046	1.007	1.086	0.140	0.118	0.162
MEHHP	1.034	1.020	1.048	**-0.002**	**-0.008**	**0.005**
MEOHP	**0.992**	**0.976**	**1.008**	**-0.002**	**-0.007**	**0.003**
MECPP	**0.994**	**0.986**	**1.003**	0.005	0.001	0.009
MCMHP	1.028	1.008	1.049	0.021	0.016	0.026
MHxP	1.037	1.012	1.062	**-0.009**	**-0.034**	**0.016**
MHHxP	0.930	0.905	0.955	**0.015**	**0.000**	**0.031**
MCPeP	0.971	0.953	0.988	**0.004**	**-0.007**	**0.015**
MCHP	**1.012**	**0.991**	**1.032**	**0.012**	**-0.004**	**0.028**
MHepP	0.922	0.880	0.965	**0.024**	**-0.010**	**0.059**
MHHpP	**0.984**	**0.964**	**1.003**	**0.006**	**-0.007**	**0.019**
MCHxP	0.960	0.935	0.984	**0.018**	**0.000**	**0.037**
MOP	1.051	1.019	1.082	**0.009**	**-0.021**	**0.039**
MCPP	1.153	1.055	1.251	**-0.008**	**-0.033**	**0.017**
MiNP	**1.039**	**0.990**	**1.088**	0.060	0.025	0.096
MHiNP	1.060	1.039	1.081	**-0.011**	**-0.030**	**0.009**
MOiNP	1.043	1.018	1.068	**-0.016**	**-0.042**	**0.009**
MCiOP	**1.003**	**0.990**	**1.015**	**0.000**	**-0.009**	**0.009**
MiDP	0.908	0.851	0.965	**0.003**	**-0.040**	**0.046**
MHIDP	1.026	1.004	1.048	**-0.005**	**-0.021**	**0.012**
MOIDP	1.049	1.012	1.085	**-0.014**	**-0.043**	**0.015**
MCINP	1.033	1.009	1.057	**-0.002**	**-0.021**	**0.017**

Confidence intervals (according to ICH algorithm) for slopes crossing 1 and intercepts crossing 0 are marked in bold.

Supplemental Table 7. Method validation: Intra-day[a] accuracy and precision of quality control materials in Q low and Q high concentrations, limit of detection (LOD)[b] and linear range[c]

	Linear range		LOD	Q Low			Q High		
				Mean	RSD	Recovery	Mean	RSD	Recovery
**Metabolite**	**ng/mL**	**R2**	**ng/mL**	**ng/mL**	**%**	**%**	**ng/mL**	**%**	**%**
MMP	0.1-100	0.9601	0.44	1.84	9.3	91.8	9.93	7.3	99.3
MEP	0.5-500	0.9998	0.65	1.87	18.2	93.3	50.29	1.5	100.6
MiPrP	0.1-100	0.9989	0.4	1.24	21.9	124.3	11.12	6.7	111.2
MPrP	0.1-100	0.9992	0.23	0.89	22.7	89.5	11.19	1.7	111.9
MiBP	0.5-500	0.9997	0.75	5.26	10.5	105.2	47.64	3.4	95.3
MnBP	0.5-500	0.9998	0.61	5.42	9.8	108.3	47.05	1.7	94.1
MHBP	0.1-100	0.9988	0.22	4.77	14.3	95.4	47.75	3.5	95.5
MBzP	0.1-100	0.9998	0.26	4.91	10.0	98.2	99.86	2.1	99.9
MPP	0.1-100	0.9987	0.27	0.75	19.3	75.0	10.99	5.2	109.9
MHPP	0.1-100	0.9980	0.38	1.12	5.6	111.8	11.53	6.7	115.3
MEHP	0.1-100	0.9998	0.74	0.72	13.7	71.8	11.19	9.2	111.9
MEHHP	0.5-500	0.9998	0.59	2.15	9.5	107.4	49.26	2.2	98.5
MEOHP	0.5-500	0.9996	0.45	1.91	6.5	95.5	48.91	1.3	97.8
MECPP	0.5-500	0.9999	0.25	1.98	3.5	99.0	47.80	1.0	95.6
MCMHP	0.1-100	0.9988	0.39	1.15	11.2	114.7	12.04	8.7	120.4
MHxP	0.1-100	0.9996	0.38	0.76	24.8	76.2	10.82	8.5	108.2
MHHxP	0.1-100	0.9982	0.26	1.10	8.5	109.6	11.33	4.9	113.3
MCPeP	0.1-100	0.9994	0.2	1.02	6.8	101.7	10.90	3.0	109.0
MCHP	0.1-100	0.9990	0.27	1.98	8.7	99.0	11.27	3.9	112.7
MHepP	0.1-100	0.9970	0.38	2.08	6.1	103.9	12.12	2.7	121.2
MHHpP	0.1-100	0.9995	0.15	1.00	10.4	100.2	11.58	0.9	115.8
MCHxP	0.1-100	0.9997	0.23	0.97	15.5	97.0	11.36	2.1	113.6
MOP	0.1-100	0.9997	0.7	2.74	25.9	136.8	11.56	10.4	115.6
MCPP	0.1-500	0.9994	0.19	11.83	12.8	118.3	53.29	9.1	106.6
MiNP	0.1-100	0.9993	0.53	5.07	24.4	101.4	9.85	13.1	98.5
MHiNP	0.1-500	0.9994	0.4	0.98	9.0	97.5	50.14	1.8	100.3
MOiNP	0.1-500	0.9997	0.31	0.92	8.0	91.7	48.78	1.2	97.6
MCiOP	0.1-500	0.9999	0.13	0.98	4.9	97.5	49.28	2.3	98.6
MiDP	0.1-100	0.9957	0.72	1.96	31.2	98.2	8.92	19.0	89.2
MHiDP	0.1-500	0.9999	0.31	0.98	8.5	98.5	49.69	1.5	99.4
MOiDP	0.1-500	0.9995	0.31	0.95	8.9	95.3	49.73	1.4	99.5
MCiNP	0.1-500	0.9999	0.32	1.02	9.9	102.1	50.74	1.0	101.5

RSD: relative standard deviation; R2: mean of five R2-values

[a] Intra-day accuracy and precision based on five repeats of quality control materials prepared as serum pool spiked at a low and a high level.

[b] LOD, limit of detection (according to ICH algorithm) based on five repeated calibration curves prepared by spiking standards in serum pool in the range 0.1-50 ng/mL.

[c] linear range (according to ICH algorithm) based on two repeated calibration curves prepared in water.

Supplemental Table 8. Method validation: Inter-day precision[a] of quality control materials in Q low and Q high concentrations

	Q Low		Q High	
	Mean	RSD	Mean	RSD
Metabolite	ng/mL	%	ng/mL	%
MMP	1.09	28.4	5.70	12.0
MEP	2.01	15.0	10.28	7.7
MiPrP	1.86	11.2	9.84	7.8
MPrP	1.97	7.5	9.85	6.9
MiBP	2.38	13.6	10.22	10.2
MnBP	2.42	15.9	10.62	6.9
MHBP	1.76	19.2	9.72	10.2
MBzP	1.98	14.4	10.23	6.0
MPP	1.76	11.8	9.45	10.7
MHPP	1.82	19.1	9.43	12.5
MEHP	2.06	19.6	10.00	10.4
MEHHP	1.89	10.7	9.94	6.0
MEOHP	1.90	9.8	9.79	5.7
MECPP	1.93	13.8	9.59	13.8
MCMHP	2.32	8.1	11.54	6.1
MHxP	1.93	5.5	9.80	5.4
MHHxP	2.06	5.3	10.15	4.8
MCPeP	2.03	6.5	10.15	5.7
MCHP	2.05	6.8	10.03	6.5
MHepP	1.85	17.5	9.63	16.1
MHHpP	2.02	4.6	10.04	4.8
MCHxP	1.91	7.4	9.55	5.7
MOP	2.11	13.4	9.99	11.2
MCPP	1.78	27.7	10.27	14.2
MiNP	1.73	26.7	9.89	14.6
MHiNP	2.01	7.9	9.96	6.1
MOiNP	1.94	9.8	9.69	9.0
MCiOP	1.97	11.0	10.28	10.0
MiDP	1.59	25.5	7.54	19.6
MHiDP	2.03	5.4	10.23	6.4
MOiDP	2.00	7.4	9.90	8.0
MCiNP	2.05	8.3	10.19	6.9

RSD: relative standard deviation

[a]Inter-day precision based on serum pool spiked with low and high amounts of standards, n=42 analyzed in 14 batches over a period of three month.

Supplemental Table 9. Pearson correlations between the ranked concentrations of maternal serum phthalate metabolites and their sums for all Raine Study participants (n=982).

	∑MBP$_{(i+n)}$	∑DEHPm	∑DiNPm	∑LMW phth.m	∑HMW phth.m	∑all phth.m	∑DEHPm+DiNPm
MEP	**0.25****	**0.16****	**0.11****	**0.75****	**0.15****	**0.66****	**0.19****
MiBP	**0.79****	0.05	**0.10****	**0.56****	**0.07****	**0.46****	0.12
MnBP	**0.92****	**0.27****	**0.16****	**0.69****	**0.26****	**0.65****	**0.29****
MEHP	**0.11****	**0.80****	0.05	**0.16****	**0.62****	**0.39****	**0.59****
MECPP	**0.20****	**0.44****	**0.26****	**0.21****	**0.39****	**0.31****	**0.47****
MCMHP	**0.16****	**0.55****	0.06	**0.16****	**0.39****	**0.29****	**0.43****
MiNP	**0.15****	**0.07****	**0.98****	**0.14****	**0.53****	**0.28****	**0.70****
MCiOP	**0.08****	**0.26****	**0.33****	**0.13****	**0.31****	**0.21****	**0.37****

**P<0.05; *P<0.10.

	MiBP	MnBP	MEHP	MECPP	MCMHP	MiNP	MCiOP
MEP	**0.19****	**0.26****	**0.09****	**0.16****	**0.10****	**0.10****	**0.13****
MiBP		**0.61****	-0.04	**0.16****	**0.11****	**0.11****	0.05*
MnBP			**0.18****	**0.21****	**0.18****	**0.15****	**0.10****
MEHP				0.03	**0.15****	0.03	0.05
MECPP					**0.67****	**0.22****	**0.54****
MCMHP						0.02	**0.33****
MiNP							**0.25****

**P<0.05; *P<0.10.

Supplemental Table 10. Pearson correlations between the ranked concentrations of maternal serum phthalate metabolites and their sums for all male participants who took part in the Raine study 20 year follow-up study (n=216).

	∑$MBP_{(i+n)}$	∑DEHPm	∑DiNPm	∑LMW phth.m	∑HMW phth.m	∑all phth.m	∑DEHPm+DiNPm
MEP	**0.22****	0.00	-0.08	**0.78****	-0.06	**0.63****	-0.06
MiBP	**0.79****	-0.03	**-0.16****	**0.49****	-0.11	**0.39****	-0.07
MnBP	**0.91****	**0.19****	-0.10	**0.60****	0.06	**0.54****	0.09
MEHP	0.05	**0.80****	0.11*	0.06	**0.61****	**0.33****	**0.61****
MECPP	0.03	**0.48****	0.12*	0.01	**0.39****	**0.23****	**0.38****
MCMHP	0.13*	**0.52****	0.08	0.13*	**0.37****	**0.30****	**0.38****
MiNP	-0.11	0.10	**0.97****	-0.10	**0.63****	**0.14****	**0.66****
MCiOP	-0.05	**0.33****	**0.27****	-0.05	**0.38****	**0.18****	**0.38****

***P*<0.05; **P*<0.10.

	MiBP	MnBP	MEHP	MECPP	MCMHP	MiNP	MCiOP
MEP	**0.14****	**0.20****	-0.06	0.02	0.09	-0.09	0.06
MiBP		**0.61****	-0.08	-0.04	0.10	**-0.17****	**-0.14****
MnBP			**0.14****	0.04	0.11	-0.11	-0.04
MEHP				0.06	0.10	0.10	**0.14****
MECPP					**0.75****	0.08	**0.56****
MCMHP						0.05	**0.37****
MiNP							**0.19****

P<0.05; **P*<0.10.
